# Mammalian target of rapamycin signaling in diabetic cardiovascular disease

**DOI:** 10.1186/1475-2840-11-45

**Published:** 2012-07-16

**Authors:** Zhao Zhong Chong, Kenneth Maiese

**Affiliations:** 1 Laboratory of Cellular and Molecular Signaling, Newark, NJ, USA; 2 Cancer Institute of New Jersey, New Jersey Health Sciences University, 205 South Orange Avenue, Newark, NJ, 07101, USA; 3 New Jersey Health Sciences University, Newark, NJ, 07101, USA

**Keywords:** Akt, AMPK, Cardiac, Diabetes Mellitus, Endothelial, Insulin Receptor Substrate 1, Sirtuin, SIRT1, TORC1, TORC2

## Abstract

Diabetes mellitus currently affects more than 170 million individuals worldwide and is expected to afflict another 200 million individuals in the next 30 years. Complications of diabetes as a result of oxidant stress affect multiple systems throughout the body, but involvement of the cardiovascular system may be one of the most severe in light of the impact upon cardiac and vascular function that can result in rapid morbidity and mortality for individuals. Given these concerns, the signaling pathways of the mammalian target of rapamycin (mTOR) offer exciting prospects for the development of novel therapies for the cardiovascular complications of diabetes. In the cardiovascular and metabolic systems, mTOR and its multi-protein complexes of TORC1 and TORC2 regulate insulin release and signaling, endothelial cell survival and growth, cardiomyocyte proliferation, resistance to β-cell injury, and cell longevity. Yet, mTOR can, at times, alter insulin signaling and lead to insulin resistance in the cardiovascular system during diabetes mellitus. It is therefore vital to understand the complex relationship mTOR and its downstream pathways hold during metabolic disease in order to develop novel strategies for the complications of diabetes mellitus in the cardiovascular system.

## Introduction

The mammalian target of rapamycin (mTOR) is a serine/threonine protein kinase that controls cellular growth as well as cellular homeostasis [1]. It was isolated in *Saccharomyces cerevisiae* with the generation of rapamycin-resistant TOR mutants that resulted in the identification of proteins participating in rapamycin toxicity with two homologous genes, namely *TOR1* and *TOR2*[2]. In eukaryotes, a single gene *TOR* is present [3]. The protein mTOR is expressed throughout the body and is present in the brain, cardiopulmonary system, gastrointestinal system, immune system, skeletal system, and the reproductive system [4]. The mTOR protein is a 289 kDa protein with multiple domains. The carboxy-terminal acid kinase domain contains a conserved sequence with homology to the catalytic domain of phosphoinositide 3 –kinase (PI 3-K) family [5]. In this domain are the regulatory phosphorylation sites of mTOR that include serine^2448^, serine^2481^, threonine^2446^, serine^2159^, and threonine^2164^[6-9]. The C-terminal also contains FKBP12 (FK506 binding protein 12) -rapamycin-associated protein (FRAP), ataxia-telengiectasia (ATM), and transactivation/transformation domain-associated protein domain (FAT) [10]. The FKBP12-rapamycin binding domain (FRB) is adjacent to the FAT domain and is the site of interaction between mTOR and FKBP12 protein bound to rapamycin [11]. The N-terminal of mTOR contains at least a 20 HEAT (Huntingtin, Elongation factor 3, A subunit of Protein phosphatase-2A, and TOR1) repeat [12]. This site provides the necessary binding of the mTOR complex for multimerization with the regulatory-associated protein mTOR (Raptor) or rapamycin-insensitive companion of mTOR (Rictor) [12]. The phosphorylation site serine^1261^ within the HEAT domain can be phosphorylated by insulin signaling both in mTORC1 and mTORC2 through PI 3-K [13]. This leads to an increase in the activity of mTOR and phosphorylation of this site also is required for mTOR serine^2481^ autophosphorylation [13].

## Signaling pathways of mTOR

**Figure 1:**
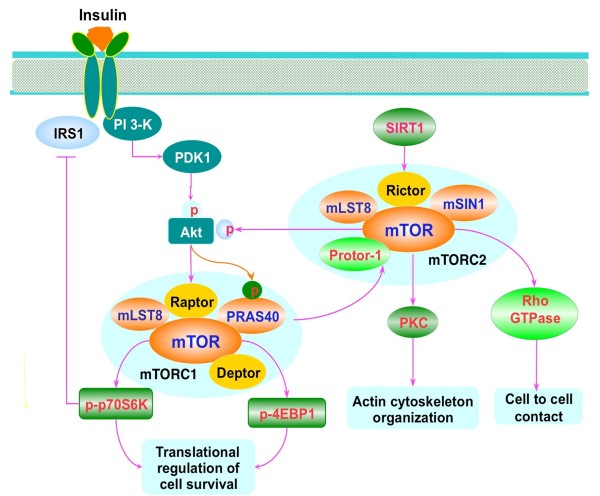
**Insulin mammalian target of rapamycin signaling pathways.** Insulin activates mTORC1 through phosphoinositide 3 kinase (PI 3-K)/Akt mediated pathways. mTORC1 consists of the regulatory-associated protein of mTOR (Raptor), the proline rich Akt substrate 40 kDa (PRAS40), the mammalian lethal with Sec13 protein 8 (mLST8), and the DEP domain-containing mTOR interacting protein (Deptor). Insulin can stimulate PI 3-K activation and subsequent recruitment of Akt to the plasma membrane through activation by phosphoinositide dependent kinase 1 (PDK1). Once active, Akt can result in the activation of mTORC1 through a series of signaling pathways. Akt can also directly phosphorylate PRAS40 and reduce its binding to Raptor and release mTORC1 from its suppression by PRAS40. Upon activation, mTORC1 phosphorylates its two major downstream targets p70 ribosome S6 kinase (p70S6K) and eukaryotic initiation factor 4E-binding protein 1 (4EBP1) and mediates cell growth, proliferation, and cell survival. mTOR can lead to inhibitory phosphorylation of the insulin receptor substrate 1 (IRS1). mTORC2 contains Rictor, mTOR, mLST8, Deptor, the mammalian stress-activated protein kinase interacting protein (mSIN1), and protein observed with Rictor-1 (Protor-1). The sirtuin SIRT1 may regulate the transcription of the gene encoding rapamycin insensitive companion of mTOR (Rictor) and promote the activation of mTORC2. mTORC2 regulates actin skeleton organization and cell survival through activating Akt and protein kinase C (PKC). In addition, mTORC2 can activate Rho GTPases and control cell to cell contact via Rho signaling pathways.

mTOR can form two multi-protein complexes that consist of mTOR Complex 1 (mTORC1) and mTOR Complex 2 (mTORC2) [1,14]. mTORC1 employs the regulatory-associated protein of mTOR (Raptor) as a scaffolding protein which is essential to recruit mTOR substrates to mTORC1 [15]. The other components of mTORC1 are the proline rich Akt substrate 40 kDa (PRAS40), the mammalian lethal with Sec13 protein 8 (mLST8), and the DEP domain-containing mTOR interacting protein (Deptor) [1,4,16]. Also known as Akt1s1, PRAS40 can block mTORC1 activity through its association with Raptor [17,18]. Insulin can stimulate the phosphorylation of PRAS40 through protein kinase B (Akt) to prevent the inhibition of mTORC1 by PRAS40 [19]. mLST8 may function to maintain insulin signaling through FoxO3 [20] and has recently been associated with extension of lifespan in mice [21]. Deptor expression is inhibited by mTORC1 and mTORC2 [1,4,16]. In the absence of Deptor, Akt, mTORC1 and mTORC2 activities are increased, but in some forms of cancer, Deptor expression is necessary for Akt signaling [22] (Figure 1).

The serine/threonine kinase ribosomal protein p70S6K and the eukaryotic initiation factor 4E-binding protein 1 (4EBP1) are two downstream targets of mTORC1. The binding of Raptor to mTOR is necessary for mTOR-catalyzed phosphorylation of 4EBP1. This binding enhances mTOR kinase activity toward p70S6K [23]. In contrast, PRAS40 can competitively inhibit the binding of the mTORC1 substrates p70S6K and 4EBP1 to Raptor. Phosphorylation of p70S6K by mTORC1 promotes mRNA biogenesis, translation of ribosomal proteins, and cell growth [24]. In the hypophosphorylated state, 4EBP1 binds competitively to the translation initiation factor eukaryotic translation initiation factor 4 epsilon (eIF4E) to block translation through eukaryotic translation initiation factor 4 gamma (eIF4G), a protein necessary to bring mRNA to the ribosome, by inhibiting contact of eIF4E with 5’- capped mRNAs. Phosphorylation of 4EBP1 by mTORC1 results in the dissociation from eIF4E to allow eIF4G to begin mRNA translation. Insulin has been found to augment phosphorylation of 4EBP1 through mTOR mediated pathways [25].

Unlike mTORC1, mTORC2 does not have Raptor as a component but contains Rictor (Figure 1). However, mTORC2 shares several common components with mTORC1, such as mTOR, mLST8, and Deptor. mTORC2 also associates with the mammalian stress-activated protein kinase interacting protein (mSIN1) and protein observed with Rictor-1 (Protor-1). mTORC2 not only regulates actin cytoskeleton organization [26], but also can control cell size, cell cycle progression [27], and endothelial cell survival and migration [28]. Rictor is relatively insensitive to rapamycin, promotes the activity of mTORC2, and is necessary for insulin-stimulated glucose uptake [29]. Rictor enables mTORC2 to phosphorylate Akt at Ser^473^ to lead to its activation and to facilitate threonine^308^ phosphorylation by phosphoinositide-dependent kinase 1 (PDK1) [30]. As previously noted, mLST8 is important to maintain the Rictor-mTORC2 interaction along with Rictor phosphorylation of Akt and PKCα [20]. Alternate splicing can generate at least 5 isoforms for mSIN1 that can respond to different signals, such as insulin, to allow TORC2 to phosphorylate Akt [31]. Rictor and mSIN1 have been shown to stabilize each other to form the structural basis of mTORC2 and are required for Akt phosphorylation [32]. Recently, mTOR has been shown to phosphorylate mSIN1 to prevent its lysosomal degradation [33]. Protor-1 is a Rictor-binding subunit of mTORC2 that does not affect the expression or activity of the other mTORC2 components to phosphorylate Akt or PKCα [34]. Protor-1 may function to activate serum and glucocorticoid induced protein kinase 1 (SGK1) through mTORC2. Loss of Protor-1 in mice leads to a reduction in the hydrophobic motif phosphorylation of SGK1 and its substrate N-Myc downregulated gene 1 in the kidney [35]. Similar to TORC1, TORC2 inhibits Deptor expression. In addition, Deptor can negatively modulate TORC2 [22].

Downstream targets of TORC2 include Akt, protein kinase C (PKC), P-Rex1 and P-Rex2, and Rho GTPases. P-Rex1 and P-Rex2 are tied to Rac activation, mTORC2, and cell migration [36]. mTORC2 also phosphorylates PKCα to modulate cytoskeleton remodeling [37], increases Akt activation [30], and affects Rho signaling pathways that control cell - cell contact [38].

## Diabetes mellitus and cardiovascular disease

Diabetes mellitus (DM) is becoming a significant burden to the world healthcare system with more than 170 million individuals affected throughout the globe [39-42]. In the Unites States alone, it is expected that greater than 20 million individuals in the United States suffer from DM with an additional significant number of individuals that are undiagnosed DM [43], signaling the need for improved healthcare for metabolic disorders [41,44,45]. Type 1 insulin-dependent DM is present in approximately 10 percent of all diabetics, is increasing in adolescent minority groups, and leads to complications in the cardiovascular system [46,47]. Patients with Type 1 DM have insulin resistance that is usually characteristic of Type 2 DM and can result in neurological and vascular disease [48,49]. Type 2 noninsulin-dependent DM represents at least 80 percent of all diabetics and represents a progressive deterioration of glucose tolerance with early β-cell compensation for insulin resistance [50,51]. This is followed by progressive decrease in β-cells mass. Type 2 DM usually occurs in individuals over 40 years of age and is increasing in incidence as a result of changes in human behavior and increased body mass index [40,44,52-54].

Complications of DM and insulin resistance have been closely tied to the release of reactive oxygen species (ROS) and subsequent oxidative stress [45,55,56]. The initial period of elevated glucose may increase the presence of potentially protective pathways [57-59], but more prolonged exposure of elevated glucose can lead to ROS generation [60,61] and can be detrimental even if glucose levels are controlled [62]. Hyperglycemia can lead to oxidative stress in the cardiovascular system [47,63]. Prolonged periods of elevated glucose is not necessary to lead to vascular oxidative stress injury, since minimal periods of hyperglycemia generate ROS and lead to endothelial cell death [64-66]. Elevated glucose in human endothelial cells also can raise the expression of antioxidants that include superoxide-dismutase, catalase, and glutathione peroxidase, illustrating that vascular cells may attempt to initially negate the effects of oxidant stress injury [67]. In addition, pathways associated with the transcriptional coactivator, peroxisome proliferator activated receptor-gamma coactivator 1α (PGC-1α), may provide protection in the cardiovascular system to maintain mitochondrial homeostasis [68]. Other therapeutic regiments that block oxidative stress in the vascular system involve the cytokine erythropoietin [56,69] and growth factors such as insulin growth factor [70]. At the clinical level, DM in the cardiovascular system can result in platelet dysfunction [71], lead to increased mortality with acute coronary syndromes [72], and result in impairments in sympathetic nervous [73].

## Diabetes and cell longevity pathways

Knowledge from clinical studies strongly suggest that acute glucose level changes can trigger oxidative stress during Type 2 DM, suggesting the need for effective and therapeutic interventions during acute and sustained hyperglycemic episodes [74,75]. In this regard. it is interesting to note that diabetic complications that can involve multiple systems of the body including the cardiovascular system also have been closely linked with pathways of cell longevity and mTOR signaling. In studies that have examined caloric restriction in male mice, genes with the greatest statistical change following caloric restriction involved those linked to the sirtuin pathways and the inhibition of mTOR signaling [76]. Sirtuins, such as SIRT1, have been shown to be protective in the cardiovascular system during DM [77-80]. SIRT1 activation can prevent endothelial senescence during hyperglycemia [81] reduce endothelial atherosclerotic lesions during elevated lipid states [82], and prevent oxidative stress injury in cardiomyocytes through p53 deacetylation and the expression of manganese superoxide dismutase (MnSOD) [83,84]. SIRT1 blocks endothelial cell apoptosis during experimental diabetes [65,85], and prevents age-related cardiac hypertrophy, apoptosis, cardiac dysfunction, and senescence marker expression in mice [86].

SIRT1 appears to be closely linked to mTOR signaling during DM. Recent work has shown that hepatic SIRT1 deficiency yields hepatic glucose overproduction, hyperglycemia, products of oxidative stress, and inhibition of the gene encoding Rictor that lead to impaired TORC2 and Akt signaling [87]. In addition, under some conditions of cell stress such as nutrient loss in other cell systems, SIRT1 may have an inverse relationship with mTOR activity [88]. Furthermore, other studies have demonstrated that inhibition of mTOR signaling can extend lifespan in mammals [21,89], provide resistance to the loss of insulin signaling [90], and prevent age –related weight gain that can generate strain upon the cardiovascular system [91].

## mTOR signaling in diabetic cardiovascular disease

**Figure 2:**
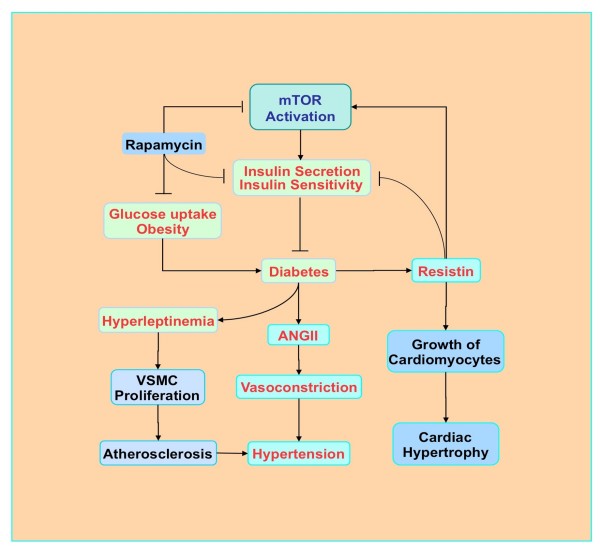
**The role of mTOR in diabetic cardiovascular disease.** Activation of mTOR promotes the secretion of insulin and increases insulin sensitivity. In contrast, rapamycin reduces insulin sensitivity, reduces glucose uptake and may prevent obesity. Hyperleptinemia can occur with diabetes and activates mTOR, stimulates vascular smooth muscle cell (VSMC) proliferation, and ultimately may contribute to atherosclerosis and hypertension. High glucose and obesity stimulate the production of angiotensin II (ANG II) to result in insulin resistance and elevated vascular tension, contributing to hypertension. Elevated resistin (for resistance to insulin) levels during diabetes can increase insulin resistance and promote mTOR activity to favor the growth of cardiomyocytes and cardiac hypertrophy.

Intact mTOR pathways may be vital for proper insulin signaling in diabetics (Figure 2. In pancreatic β-cells, loss of mTOR signaling with p70S6K has been shown to lead to hypoinsulinemia, glucose intolerance, insulin insensitivity to glucose secretion, and a decrease in β-cell size [92]. Furthermore, increased phosphorylation of p70S6K and 4EBP1 in pancreatic β-cells results in mice improved insulin secretion and resistance to β-cell streptozotocin toxicity and obesity [93]. In contrast, mTOR inhibition during rapamycin application leads to insulin resistance, reduces β-cell function and mass, limits insulin secretion, and results in DM [94]. Although inhibition of mTOR with rapamycin reduces food intake and prevents fat-diet induced obesity in mice, rapamycin administration attenuates glucose uptake and metabolism in skeletal muscle through prevention of insulin generated Akt activation and alteration in the translocation of glucose transporters to the plasma membrane [95].

It is important to note that although insulin is a potent activator of mTOR through Akt regulatory pathways, mTOR may have a negative feedback loop and led to glucose intolerance through inhibition of the insulin receptor substrate 1 (IRS1). For example, mTOR signaling through the tuberous sclerosis complex (TSC1, hamartin/ TSC2, tuberin) can inactivate IRS and phosphorylate p70S6K to block IRS1 activity by direct phosphorylation [96]. As a result, activation of the mTOR pathway in the cardiovascular system may lead to poor insulin signaling and insulin resistance. In experiments with high fat fed obese rats, activity of the mTOR pathway is elevated in skeletal muscle and leads to inhibitory phosphorylation of IRS1, impaired Akt signaling, and insulin resistance [97]. Increased consumption of high fat diets also activates the renin-angiotensin-aldosterone system with increased circulating angiotensin II (ANG II). In aortic endothelial cells, ANG II can stimulate mTOR and p70S6K activation that phosphorylates IRS1 and inhibits endothelial nitric oxide synthase that not only may contribute to insulin resistance but also to vasoconstriction and hypertension [98].

Cardiac protection during DM may rely upon the activation of AMP activated protein kinase (AMPK). AMPK can phosphorylate tuberin (TSC2) and inhibit mTORC1 [99]. Increased AMPK activation has been shown to reduce myocardial infarct size during models of DM [100]. In addition, loss of AMPK activity can increase insulin resistance in skeletal muscle [101]. Down-regulation of the AMPK pathway also may be detrimental to cardiac tissue. For example, the liver kinase B1 (LKB1) can regulate the activation of AMPK via phosphorylation [102]. Loss of LKB1 has been shown to impair cardiac function during either aerobic or ischemic conditions [103], illustrating the importance of AMPK signaling in the mTOR pathway for the cardiovascular system.

Cardiac hypertrophy also may be a product of increased mTOR activity during diabetes (Figure 2). The signaling molecule resistin (for resistance to insulin) has enhanced circulating levels during obesity and diabetes. In addition, application of anti-resistin antibodies improves blood glucose and insulin efficacy in murine models of diet-induced obesity [104]. Resistin has recently been shown in rat ventricular myocytes to inhibit AMPK activity, activate TCS2 of the mTOR pathway, and increase cell size leading to cardiac hypertrophy [105]. Resistin also phosphorylates IRS1 through mTOR to promote insulin resistance [105].

Cardiovascular disease also may be mediated through hyperleptinemia and the activation of mTOR (Figure 2). Hyperleptinemia can co-exist with DM and has been shown to enhance mTOR activity and stimulate vascular smooth muscle cell proliferation [106]. Complications of drug eluting stents coated with the mTOR inhibitor rapamycin that lead to re-stenosis in patients with DM are believed to occur as a result of concurrent hyperleptinemia that can override the mTOR inhibition of rapamycin in these patients. Interestingly in patients with metabolic syndrome that have elevated insulin levels, lymphocytes of these patients have reduced expression of mTOR that may contribute to increased risk for vascular thrombosis [107]. Furthermore, exercise in obese rats increased the ability of insulin to phosphorylate Akt and led to increases in Raptor, p70S6K, and 4EBP1 phosphorylation, suggesting that under some circumstances a balanced level of mTOR pathway activity may be beneficial for patients with DM [108].

## Conclusions

Metabolic cardiovascular disease is closely regulated through the mTOR signaling pathways. The mTOR multi-protein complexes of TORC1 and TORC2 can oversee insulin signaling, vascular survival, and cardiomyocyte growth. Downstream, pathways tied to mTOR pathway components, such as p70S6K and 4EBP1, can regulate insulin secretion and resistance to β-cell injury that ultimately affect diabetic complications in the cardiovascular system. Interestingly, new studies have linked mTOR signaling to the cell longevity pathways of SIRT1 that can also provide robust cardiovascular protection against models of experimental diabetes.

Although mTOR can be beneficial to promote insulin signaling through regulatory pathways that involve Akt, a careful modulation of mTOR activity may be necessary for the treatment of cardiovascular complications during DM. Increased physical activity, which is a requisite therapy for diabetics, can promote phosphorylation of mTORC1 components Raptor, p70S6K, and 4EBP1 to assist with insulin signaling. Yet, mTOR may have a negative feedback loop and can inactivate IRS that may lead to poor insulin signaling and insulin resistance in the cardiovascular system. In addition, mTOR signaling during DM may result in cardiac hypertrophy, promote some of the ill effects of hyperleptinemia, and further diabetic retinopathy [109] given the ability of mTOR to promote angiogenesis [1,110]. Activation of pathways of mTOR also may promote tumor growth [38,111] and increase the activation of inflammatory cell pathways [112-116] that may negatively impact the cardiovascular system. It therefore becomes crucial to elucidate the varied pathways of mTOR signaling and the role that these pathways have upon the metabolic and cardiovascular systems for the effective and safe development of promising therapies that can be targeted against diabetic complications in the cardiovascular system.

## Abbreviations

AMPK, AMP activated protein kinase; ANG II, angiotensin II; ATM, ataxia-telengiectasia; Deptor, DEP domain-containing mTOR interacting protein; DM, diabetes mellitus; 4EBP1, eukaryotic initiation factor 4E-binding protein 1; eIF4E, eukaryotic translation initiation factor 4 epsilon; eIF4G, eukaryotic translation initiation factor 4 gamma; FRAP, FKBP12 (FK506 binding protein 12) -rapamycin-associated protein; FRB, FKBP12-rapamycin binding domain; IRS1, insulin receptor substrate 1; LKB1, liver kinase B1; mLST8, mammalian lethal with Sec13 protein 8; mSIN1, mammalian stress-activated protein kinase interacting protein; mTOR, mammalian target of rapamycin; MnSOD, manganese superoxide dismutase; mTORC1, mTOR Complex 1; mTORC2, mTOR Complex 2; NAD+, nicotinamide adenine dinucleotide; PGC-1α, peroxisome proliferator activated receptor-gamma coactivator 1α; PI 3-K, phosphoinositide 3 –kinase; PDK1, phosphoinositide-dependent kinase 1; PKC, protein kinase C; Akt, protein kinase B; PRAS40, proline rich Akt substrate 40 kDa; Rictor, rapamycin-insensitive companion of mTOR; ROS, reactive oxygen species; Raptor, regulatory-associated protein mTOR; SGK1, serum and glucocorticoid induced protein kinase 1; TSC1 hamartin/TSC2, tuberin, tuberous sclerosis complex.

## Competing interests

The authors have no competing interests.

## Authors’ contributions

ZZC and KM drafted the manuscript. ZZC and KM completed the final versions of the manuscript with the incorporation of figures and tables. All authors read and approved the final manuscript.
